# A real-time correction method for sample drift in STXM based on Fourier transform image registration

**DOI:** 10.1107/S1600577526006429

**Published:** 2026-07-28

**Authors:** Yuchen Jiao, Zijian Xu, Tianxiao Sun, Yufei Zhang, Yao Luo, Zhi Guo, Yong Wang, Xiangjun Zhen, Jian He, Renzhong Tai, Xiangzhi Zhang

**Affiliations:** a Shanghai Institute of Applied Physics Chinese Academy of Sciences Shanghai201800 People’s Republic of China; b University of Chinese Academy of Sciences Beijing100049 People’s Republic of China; c College of Electronic Science and Technology National University of Defense Technology Changsha410073 People’s Republic of China; d Shanghai Synchrotron Radiation Facility, Shanghai Institute for Advanced Study Chinese Academy of Sciences Shanghai201204 People’s Republic of China; Brazilian Synchrotron Light Laboratory, Brazil

**Keywords:** STXM, stack scan, online drift correction method, synchrotron radiation

## Abstract

Scanning transmission X-ray microscopy (STXM) has high-resolution imaging capabilities of tens of nanometres by using a Fresnel zone plate (FZP) to focus the X-rays. It is an important technique for synchrotron radiation experiments. Combined with energy scanning across an X-ray absorption edge of the element of interest, STXM provides a nanoscale chemical analysis capability for samples. This scan mode is also called stack scan. During stack scan, to obtain two-dimensional high-resolution imaging, samples should be on the FZP focal plane, which varies with energy, as the FZP focal length is proportional to the photon energy. The stack mode of STXM can automatically adjust the ZP position in the beam direction with respect to the FZP focal length. Due to thermal drift of the motor and misaligning of the X-ray beam path with respect to the sample plane, the scanning area of the sample can drift continuously during a stack scanning, resulting in an increased scanning area and an extended scan time. In this paper, an online drift correction method for stack scan is proposed by combining a Fourier transform image registration algorithm with the position information of a laser interferometer. With this method, the sample drift of a 100-image stack scan is reduced from over 1 µm to less than 120 nm in STXM. Therefore, the sample drift in stack scan is effectively eliminated and redundant scan area is greatly reduced, which improves experimental efficiency.

## Abstract 2

This study proposes an online drift-correction method for scanning transmission X-ray microscopy that integrates Fourier-transform image registration with laser-interferometer feedback, suppressing sample drift in a 100-image stack scan from over 1 mm to below 120 nm and thereby eliminating redundant scan area while markedly enhancing experimental throughput.

## 1. Introduction

**Figure 1:**
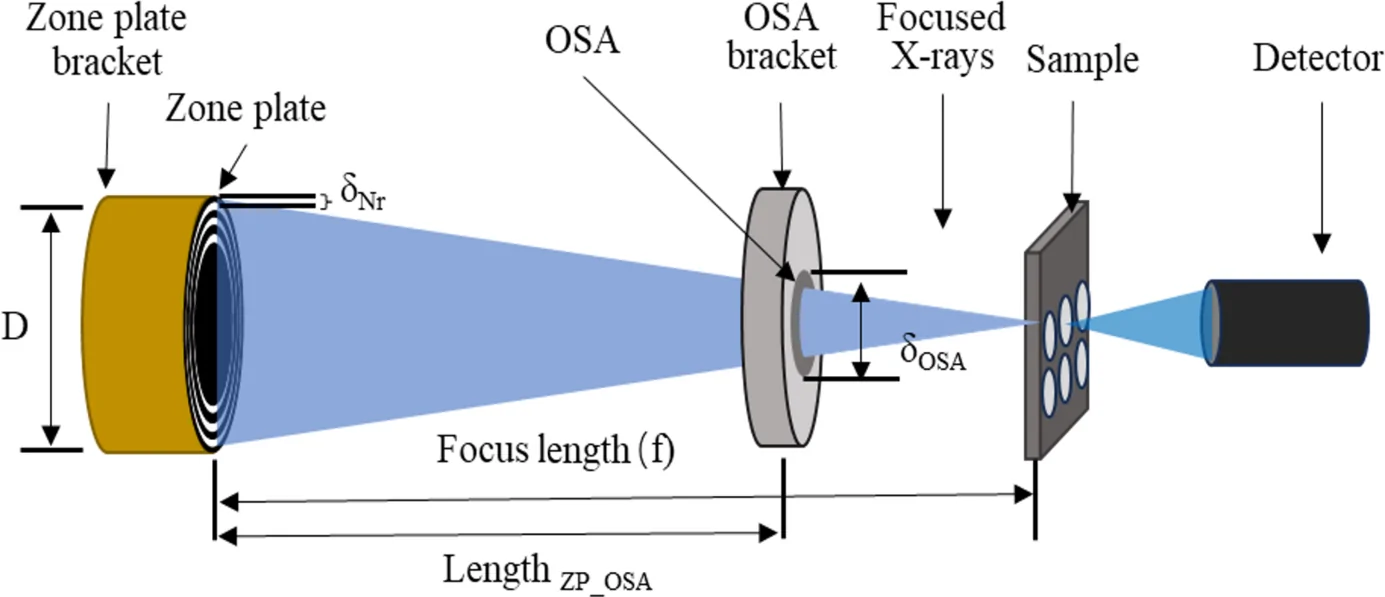
Schematic of the main optical components of the STXM device.

Scanning transmission X-ray microscopy (STXM) (Kirz *et al.*, 1992 ▸; Wang & Li, 2022 ▸; Sun *et al.*, 2021 ▸) is an important synchrotron radiation based technique, and has been widely used in biology (Bernard *et al.*, 2009 ▸; Liu *et al.*, 2013 ▸), chemistry (Nilsson *et al.*, 2005 ▸; Zhang *et al.*, 2023 ▸; Yan *et al.*, 2021 ▸), materials (Park *et al.*, 2022 ▸; Jun *et al.*, 2023 ▸), environmental (Foetisch *et al.*, 2022 ▸; Xia *et al.*, 2020 ▸; Nho *et al.*, 2014 ▸) and other fields. The setup of the STXM device is shown in Fig. 1 ▸, including a zone plate (ZP), order-selecting aperture (OSA), sample, detector and their moving stages. In this setup, monochromatic X-rays are focused to tens of nanometres by the ZP and the OSA. The sample is scanned with high-precision motors and an image at nanoscale resolution can be obtained. Combined with X-ray absorption spectroscopy scans, STXM can provide chemical state mappings of a sample with a spatial resolution of tens of nanometres (Xu *et al.*, 2024 ▸; Remusat *et al.*, 2012 ▸; Plouviez *et al.*, 2024 ▸). This scan mode, called stack scan, involves scanning the same sample area at different energies around an X-ray absorption edge of the element of interest (Shin *et al.*, 2018 ▸; Pacold *et al.*, 2016 ▸).

As is well known, first-order X-rays focused by ZP are used in STXM imaging. The focal length *f* and the distance between the OSA and the ZP are calculated using
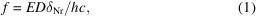

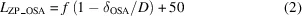
where *D* is the ZP diameter, *δ*_Nr_ is the outermost zone width of the ZP, *E* is the incident X-ray energy, *h* is the Planck constant, *c* is the speed of light, and δ_OSA_ is the OSA diameter. To avoid cutting the X-ray cone, the OSA is generally moved 50 µm further downstream. As indicated by the two equations, the ZP focal length as well as the ZP–OSA distance will change linearly with X-ray energy (Carnal *et al.*, 1991 ▸).

STXM at the Shanghai Synchrotron Radiation Facility (SSRF) adopts a bidirectional scanning method (Sun *et al.*, 2021 ▸) which uses a laser interferometer (LIM) to record positions and a photomultiplier tube to record X-ray intensity. An FPGA(field-programmable gate array) is used to control them synchronously. During a stack scan experiment, typically tens to low hundreds of 2D STXM images were collected at energies near an absorption edge of an element of interest (Dindault *et al.*, 2022 ▸; Park *et al.*, 2024 ▸) with an energy step of 0.2 to 0.5 eV and an energy range of 15 to 30 eV.

**Figure 2:**
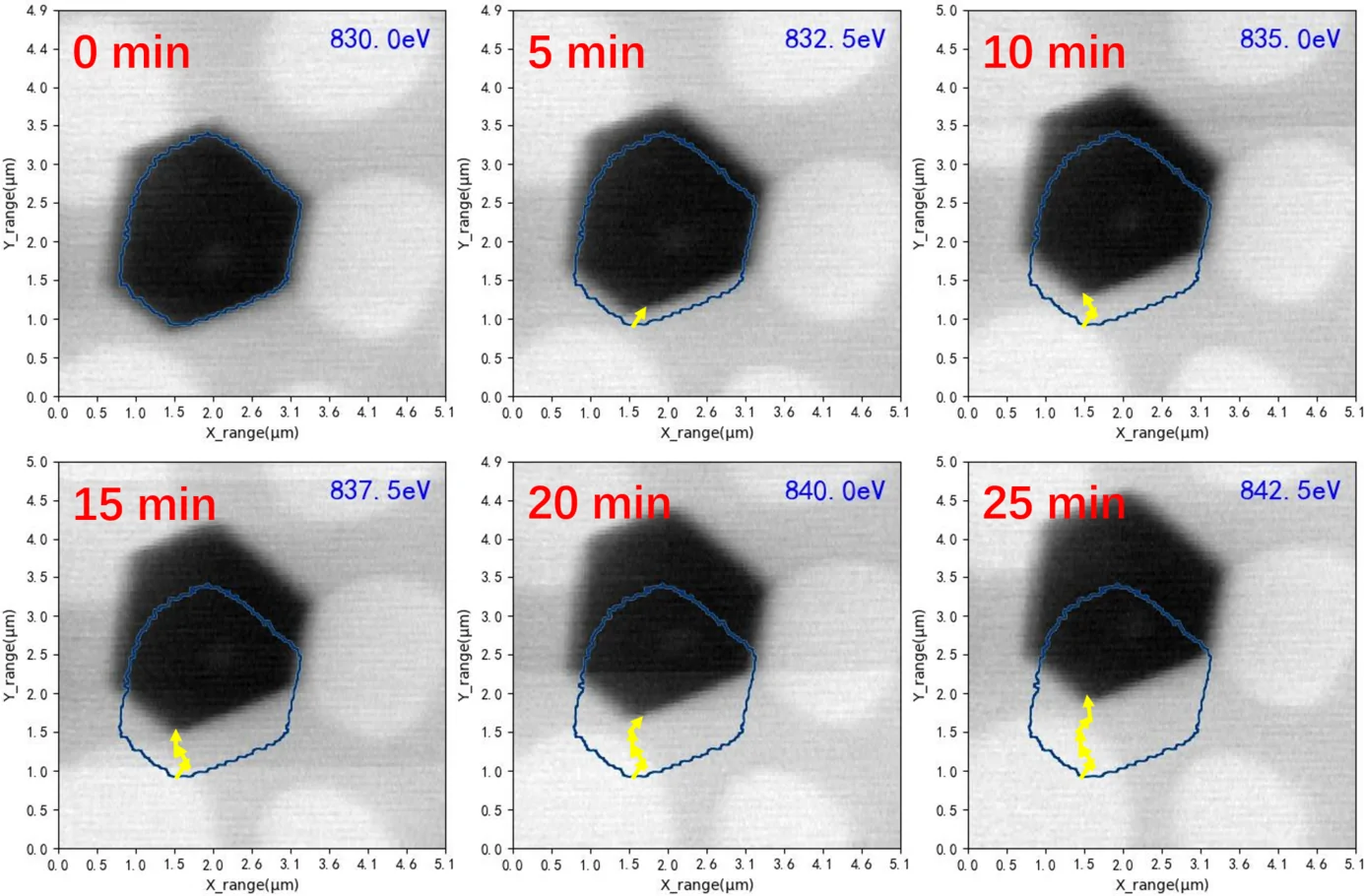
Sample drift in a stack scan with the energy ranging from 830 eV to 842.5 eV. The dark-blue circle is the profile of the sample particle in the first image.

In an STXM device, the X-ray beam path is typically not completely perpendicular to the sample plane. This misalignment combined with the thermal drift of motors results in a change of the sample imaging region with X-ray energy, which is also called sample drift. Experimental data from the Advanced Light Sources(Marcus, 2023 ▸) also shows sample drift in the stack scan process, which is about 5% between the 93rd and the first images. Fig. 2 ▸ shows partial STXM images of a stack scan between 830 eV and 842.5 eV with a step size of 0.5 eV at the BL08U1A beamline of SSRF. Each image is superimposed with a contour of a particle in dark blue. We can see that, in the process of stack scan, the sample will obviously move with the X-ray energy changing or with the ZP moving in the beam direction. Table 1 ▸ shows the offset of the sample particle in the *X* and *Y* directions in the image series shown in Fig. 2 ▸. It can be seen that there is an ∼2 µm offset of the sample between the 26th and the first images. Taking Fig. 2 ▸ as an illustrative example, with identical scanning step sizes and negligible sample offset, the scanning range can be narrowed from 5 µm × 5 µm to 4 µm × 4 µm, reducing the scanning time for an individual stack by approximately 25% and markedly improving experimental efficiency.

To ensure that no sample topography is lost by sample drifting, one has to expand the scanning range and extract the common area of the image stack in data post-processing, which seriously decreases the experiment efficiency. A common solution to the thermal drift problem is to fill the STXM chamber with helium gas to accelerate heat diffusion from motors (Nho *et al.*, 2014 ▸). An online correction method with a LIM has been used to record the position changes and compensate for them by moving the piezo-stage (Zhang, 2010 ▸). However, this method cannot completely correct the sample drift tendency, and µm-scale drifts still exist with this method.

To solve this problem, we propose an online drift correction method based on the Fourier image registration (FIR) algorithm and a LIM. By FIR between each image of the stacking process with the first one, the sample offset can be obtained in 100 ms. Occasionally, the FIR method may fail. In this situation, the position offset is measured by the LIM which provides a low-precision sample drift. Then, the sample drift is corrected before the next scan starts by moving the piezoelectric motors. As a result, the total offset of 100 stack images can be reduced from micrometres to about 120 nm without reducing the stack scan speed.

## 2. Experimental principle and design

### 2.1. Analysis of the reasons for sample drift

It is impossible to achieve complete perpendicularities between the ZP and the X-ray beam. The focused X-ray beam passing through the ZP is not absolutely perpendicular to the sample plane, and there is a small deflection angle between the X-ray beam and the normal line of the sample plane (to be precise, the movement plane of the sample scan) (Zhang, 2010 ▸) which will cause the sample image drift when the sample is stack-scanned. At the same time, the temperature changes of the sample and ZP stages will also cause position changes of the sample or the focusing spot. During a stack scan, these changes will appear as a shift of the center position of the sample image.

**Figure 3:**
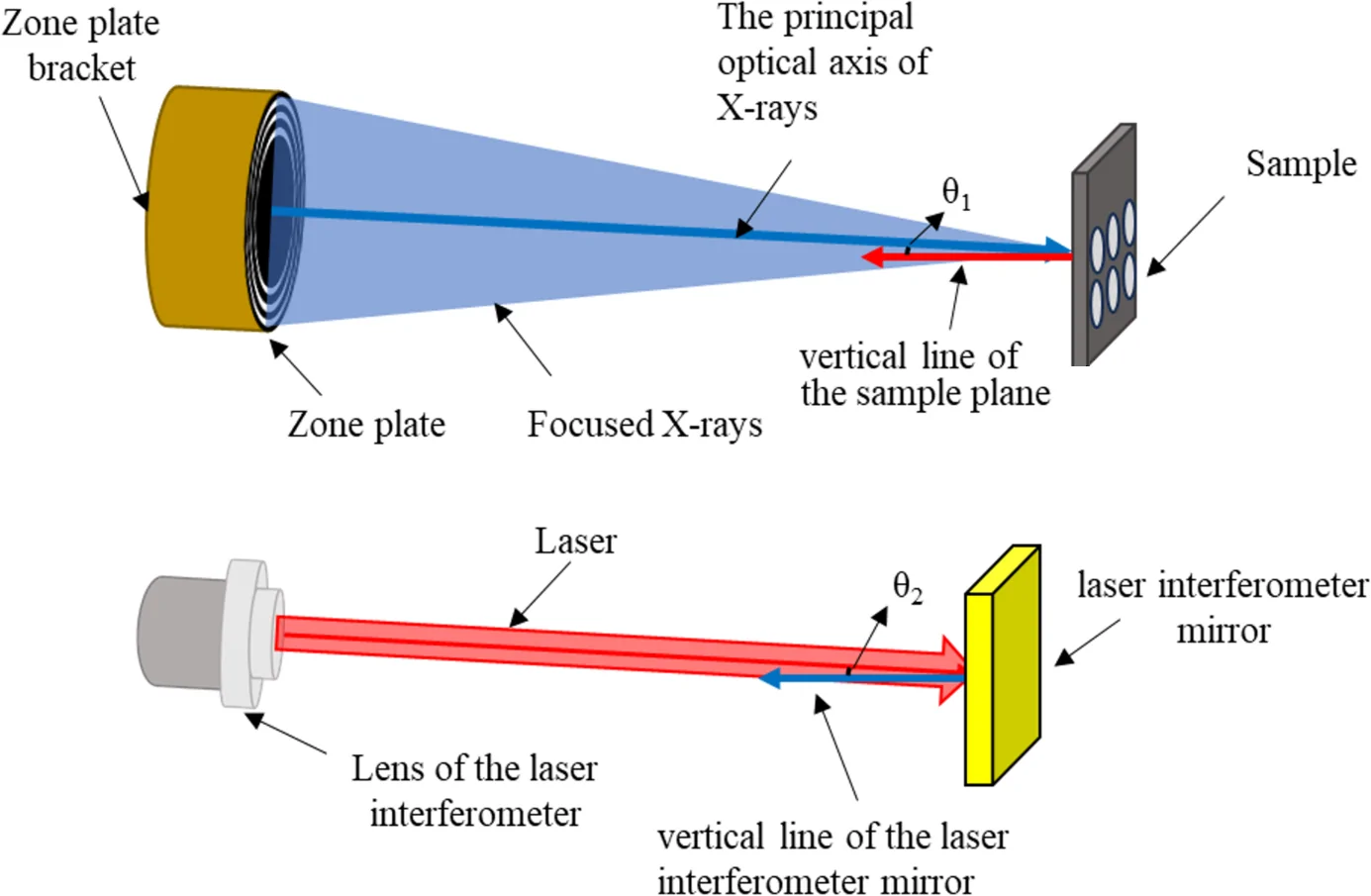
Schematic diagrams of misalignments of the X-ray beam (top) and laser (bottom) in STXM.

In an STXM endstation, a LIM is deployed between the ZP and the sample stage to monitor their relative position change (Xue *et al.*, 2010 ▸). In theory, it can effectively correct the thermal drift of the sample stage. However, as shown in Fig. 3 ▸, due to installation inaccuracies, in addition to the angle θ_1_ between the X-ray optical axis and the normal line of the sample plane, there exists another small angle θ_2_ between the laser path of the LIM and the normal of the sample movement plane. Because of these two angles, the direct use of the LIM position data cannot correct the thermal drifts of motors accurately enough, so the drifting effects of the two angles should also be corrected.

**Figure 4:**
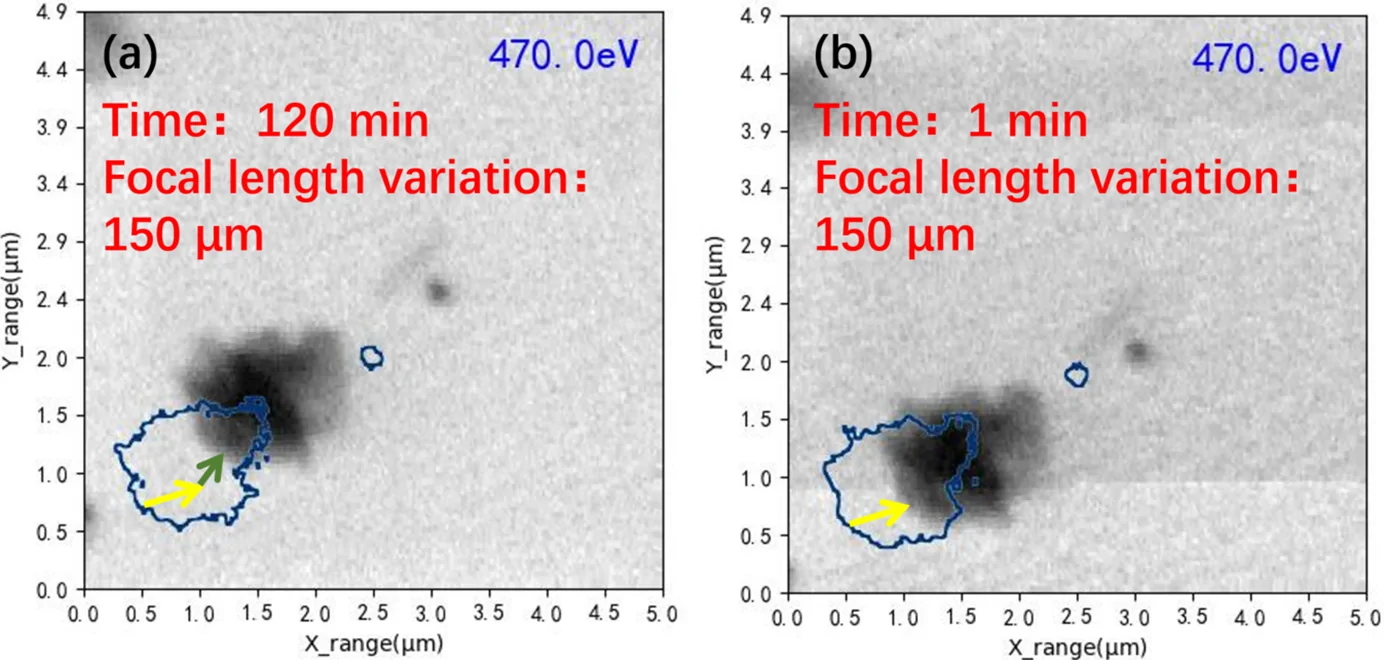
Sample drifts of two stack scans with different energy step sizes. (*a*, *b*) The last images of the two stacks with a blue contour representing the sample position in the first image. (*a*) The sample drift with a 0.3 eV step size. (*b*) The sample drift with a 30 eV step size.

To directly show the effects of sample drift caused by the above two reasons, we analyzed two stack scan results with an energy step size of 0.3 eV and 30 eV, respectively. The energy range is 440–470 eV. Since the temperature change of motors can be ignored over a short time, the sample drift in the stacking with a 30 eV step can be considered to be mainly caused by the X-ray direction misalignment (not perpendicular) with respect to the sample. In contrast, the sample drift with 0.3 eV energy steps is caused by both the focused X-ray non-perpendicularity to the sample and the thermal drift of motors. The sample drift due to thermal drift can be obtained by taking the difference between the final sample particle positions of the two stacks. Fig. 4 ▸ shows the final images from the two stacks, which are superimposed with the sample profile from the first image (the dark-blue circles in each image).

The sample drift results of the above two stack scans are shown in Table 2 ▸. It can be seen that the sample offset in the *Y* direction is caused by both X-ray non-perpendicularity and motor thermal drift, while the sample offset in the *X* direction is mainly caused by X-ray non-perpendicularity. As equation (1) shows, the higher the energy, the longer the focal length. In cases involving high X-ray energy, misalignment of the X-ray direction to the sample plane will worsen the sample drift. Therefore, it is necessary to correct the sample drift online during a stack scan experiment.

### 2.2. Rough correction scheme based on LIM

In STXM, it can be assumed that both the sample position deviation in an image and the position deviation recorded by the LIM change linearly with the energy of stack scan. Therefore, the LIM-recorded position offset (hereafter referred to as the LIM offset) is also proportional to the sample drift in the image. As a result, the sample drift can be calculated and corrected online based on the LIM offset during an experiment. To confirm this, we conducted a stack imaging of 40 energy points for a 2 µm circular particle sample. In this stacking, the energy step is 0.5 eV and the energy range is far away from any absorbing edge of this sample.

**Figure 5:**
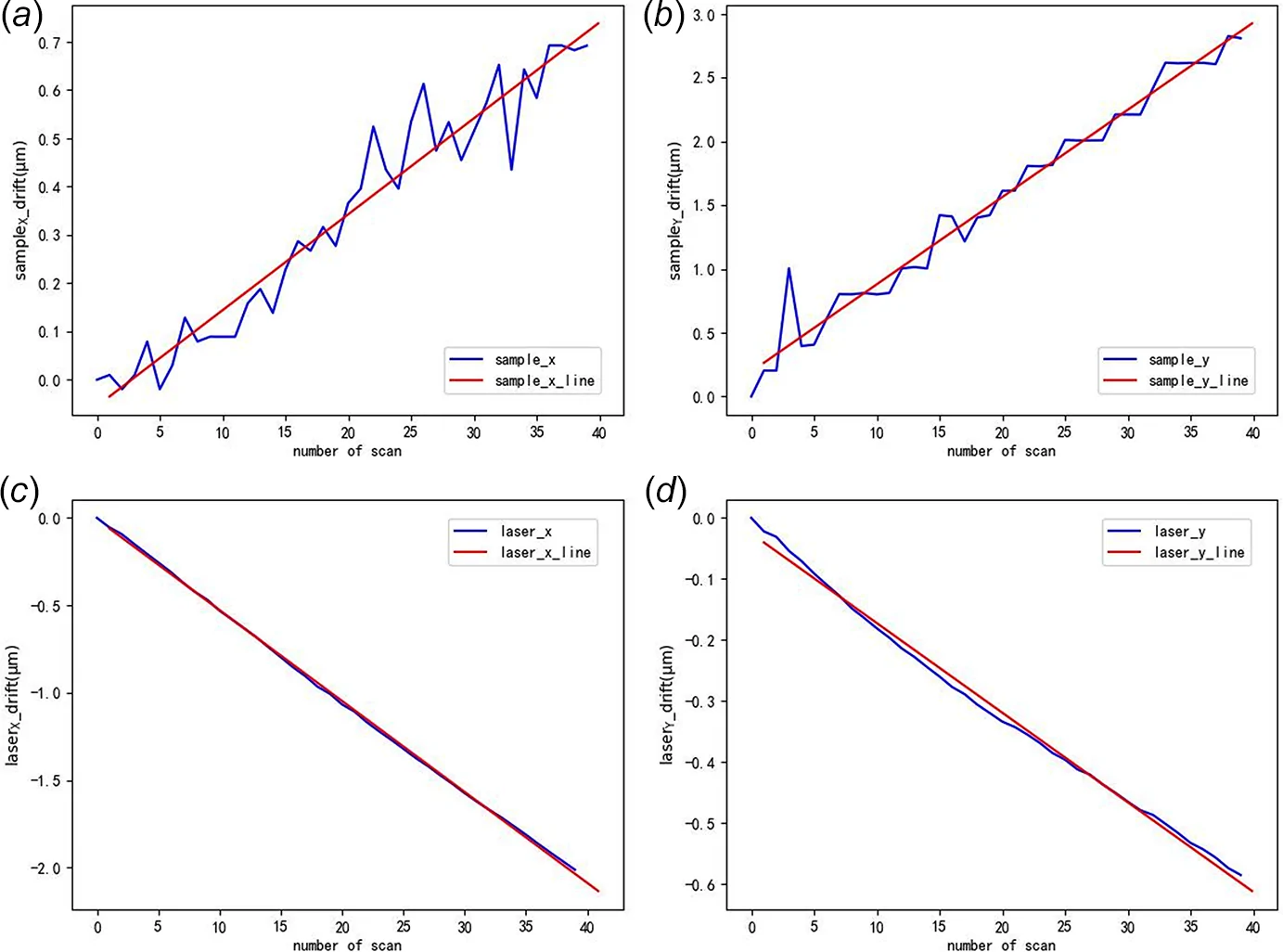
Sample drifts and LIM offsets of a stack scan with 40 energy points for a circular particle sample. The blue lines represent experimental data, while the red lines are linear fitting results. Sample drifts in (*a*) *X* and (*b*) *Y* directions. LIM offsets in (*c*) *X* and (*d*) *Y* directions.

We recorded the center position change of the particle in each image as well as the LIM offset. Here, as a preprocessing step, the particle position changes were obtained by Fourier image registration calculation as introduced in the following section. The particle position change is actually the sample drift during a stack scan. To prevent the sample drifting out of the field of view (10 µm × 10 µm) at the beginning of the experiment, the particle was placed in the lower left area of the imaging window, *i.e.* in the opposite direction to the drift. The sample drifts and LIM offsets and their linear fitting are shown in Fig. 5 ▸. These data verified the linear relationship between the LIM offset and the sample drift.

According to the results shown in Fig. 5 ▸, we obtained the*X*-direction drift equations,
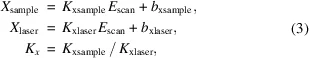
where *X*_sample_ and *X*_laser_ represent the sample drift and the LIM offset in the *X* direction, respectively; *K*_xsample_ and *K*_xlaser_ are slope coefficients of the linear fitting of the sample drifts and the LIM offsets in the *X* direction, respectively; *b*_xsample_ and *b*_xlaser_ are the corresponding intercepts of the linear fitting; *E*_scan_ is the energy change in stack scanning; *K*_*x*_ is the proportional coefficient of *K*_xsample_ over *K*_xlaser_.

Then, the sample drift in the *X* direction can be corrected using the LIM offset,
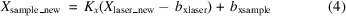
where 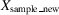 and 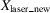 represent the new sample drift and the new LIM offset in the *X* direction, respectively. The *Y* direction drift can also be corrected in this way.

However, as the sample drift fluctuates up and down, the linear relationship between the sample drift and the LIM offset is not fully satisfied. Therefore, this method can only obtain low-precision sample drifts and only roughly correct for the drifts.

### 2.3. Fine correction scheme based on Fourier image registration (FIR)

**Figure 6:**
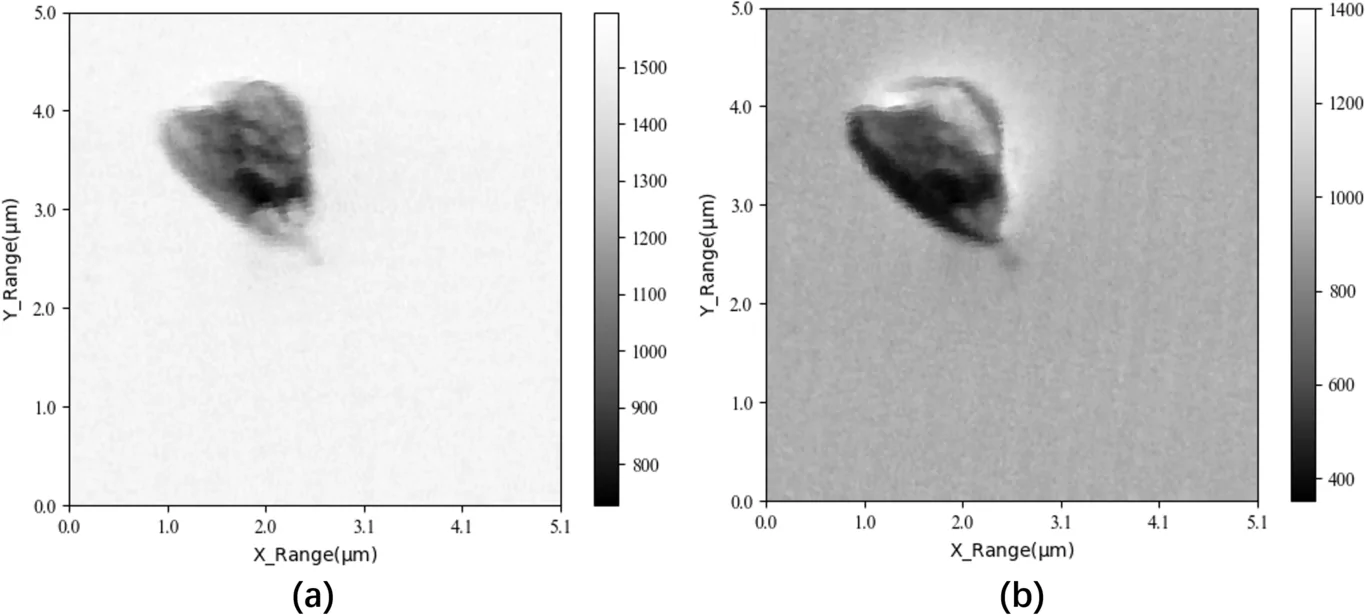
Stack images of nickel particles at two different energies. (*a*) At a non-absorption-edge energy. (*b*) At an absorption peak energy.

In order to achieve more accurate sample offsets, we adopted the Fourier-transform-based cross correlation method to register images (Tong *et al.*, 2019 ▸; Guizar-Sicairos *et al.*, 2008 ▸; Guo *et al.*, 2005 ▸). The principle is that, when an image is shifted, the cross correlation between the original and shifted images is calculated in Fourier space based on the Fourier convolution theorem; then, the cross correlation function is inversely Fourier transformed to real space and the sample drifts, Δ*x* and Δ*y*, are obtained by locating the peak of the cross correlation. For example, if the second image f2(*x*, *y*) is obtained by translating the first image f1(*x*, *y*) by (Δ*x*, Δ*y*), then we have
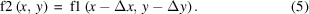
Fourier transforming the two images, the cross correlation function in Fourier space can be obtained,
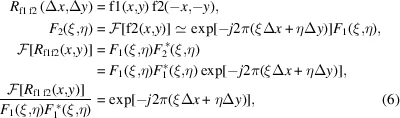

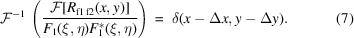
For STXM images with basically constant morphology, this method works well for calculating sample offsets at even the sub-pixel level (Tong *et al.*, 2019 ▸; Guo *et al.*, 2005 ▸). However, in stack scan, there may be obvious contrast and morphology differences between the absorption-peak and non-absorption peak images. Fig. 6 ▸ shows two images of nickel particles at an absorption edge and non-absorption-edge energies, respectively, exhibiting significant differences between the two images due to different X-ray absorptions.

**Figure 7:**
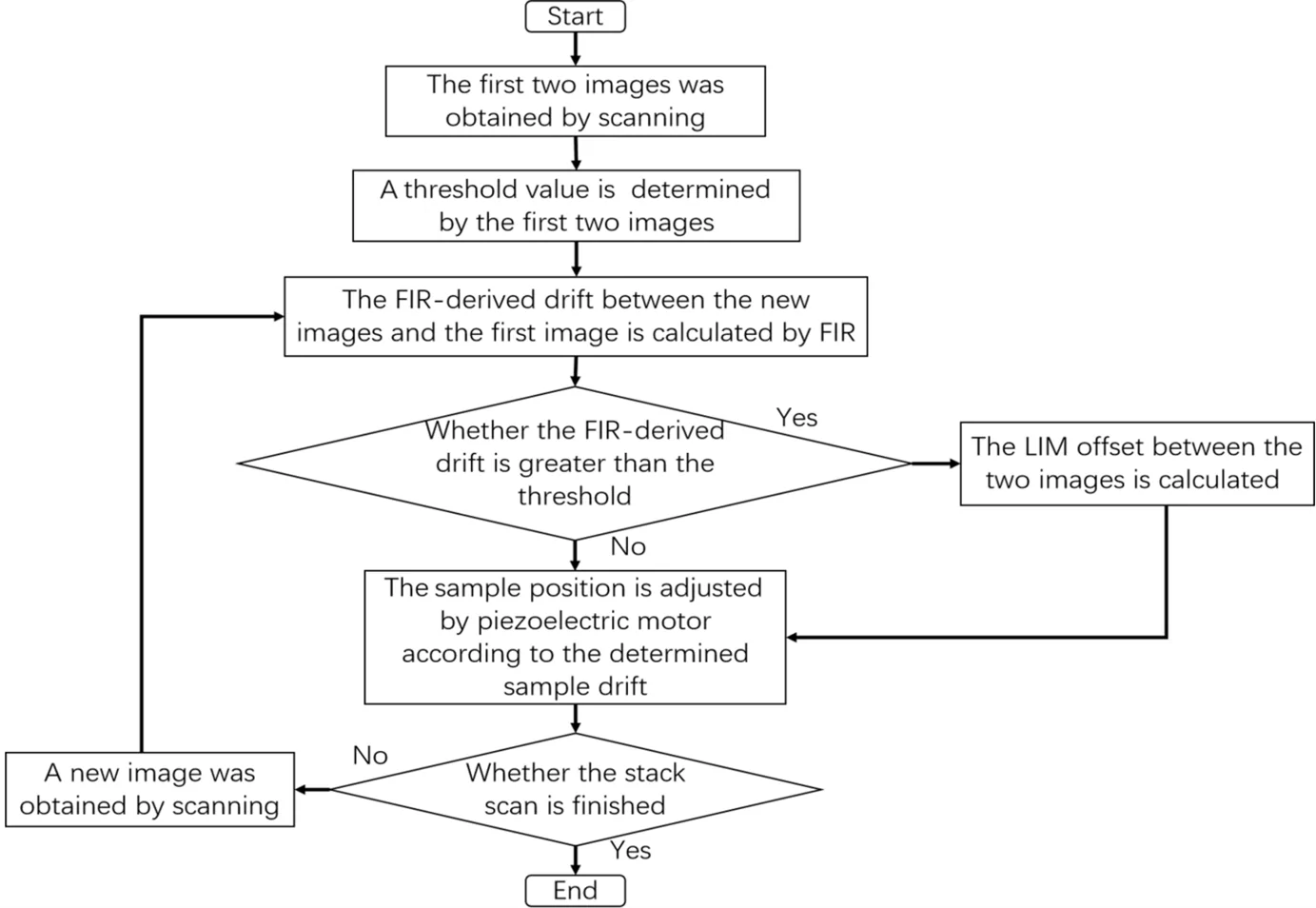
Flow chart of the integrated online correction method for stack scan drifts.

Because of the morphology and contrast differences between stack images, there may be errors in the sample drift calculation by FIR. Therefore, we propose an integrated online drift correction method for stack scan. In this method, a threshold value is first determined by doubling the sample offset of the first two images in the stack. Then, in the stacking process, if the sample drift calculated by FIR is greater than the threshold, the LIM offset will be used to correct the sample position with a lower precision; otherwise, the FIR-derived drift will be used to correct the sample position. Finally, the sample position is adjusted by piezoelectric motors according to the determined sample drift. A flowchart of the online correction method is shown in Fig. 7 ▸.

## 3. Experimental results

### 3.1. Rough correction results by LIM offsets

**Figure 8:**
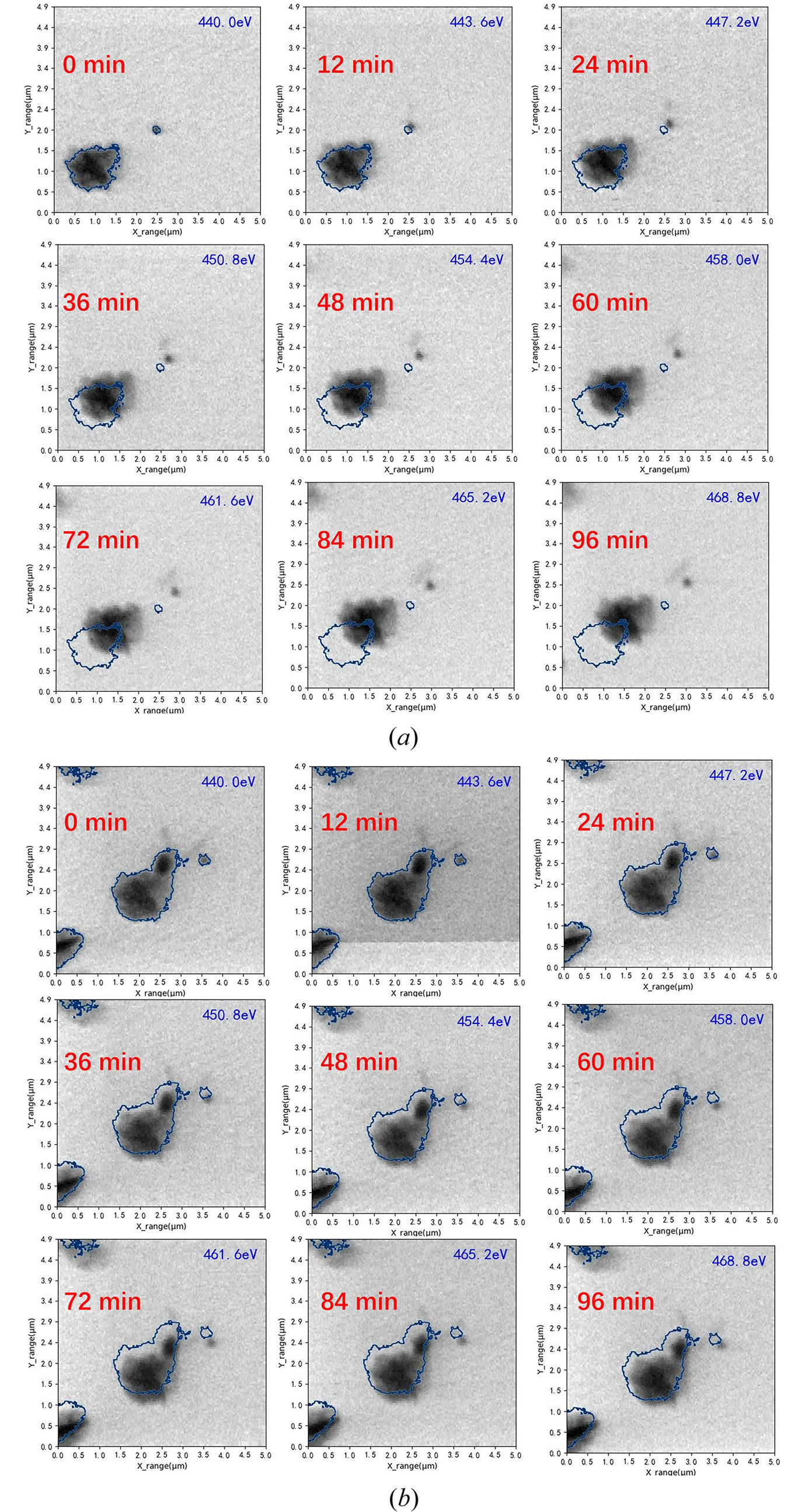
Stack scan results without correction (*a*) or with coarse correction using LIM offsets (*b*). The sample comprises particles containing Ti, and the dark-blue outline denotes the sample position in the first image of each stack.

To verify the actual effect of the LIM correction scheme, a particle sample containing element Ti was stack-scanned to collect 100 images with an energy step of 0.3 eV in the range 440–470 eV (around the *L*-edge of Ti). The stacking was performed in two modes: without correction or with coarse correction by LIM. Nine resulting images were taken from each stack with an energy interval of 3.6 eV and shown in Fig. 8 ▸. The particle contour (blue) of the first image was superimposed on each resulting image to visualize the sample drift and correction effects in the stack scan.

After calculating the position offsets using FIR, the sample drifts of the nine energies without correction and with rough correction are listed in Table 3 ▸, respectively. Table 3 ▸ shows that, without correction, the maximum sample drift is about 1.1 µm in both *X*/*Y* directions, and the drift gradually increases with the energy change. Table 3 ▸ shows that, with coarse correction, the maximum offset is below 500 nm in both *X*/*Y* directions, and the residual drift tends to first increase then decrease with the energy change. In addition, Table 3 ▸ shows that the LIM correction is better in the *X* direction than in the *Y* direction, which is consistent with the above analysis that the *X*-direction offset is mainly caused by the X-ray misalignment to the sample while the *Y*-direction offset is caused by both the X-ray misalignment and the motor thermal drift.

### 3.2. Fine correction results by the integrated online drift correction method

**Figure 9:**
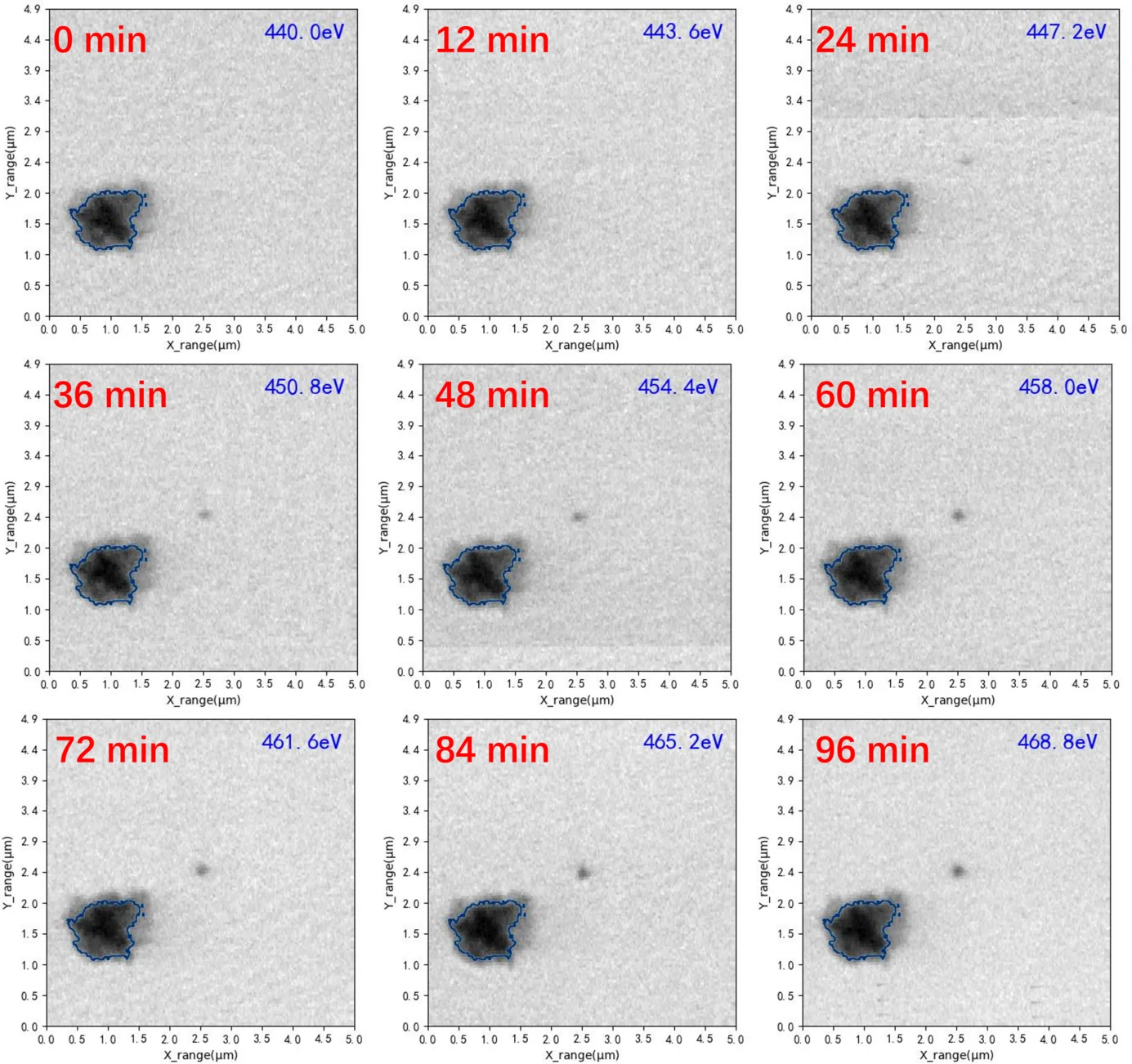
Stack scan results with fine correction of sample drifts by the integrated online correction method. The sample comprises particles containing Ti, and the dark-blue outline represents the sample position in the first image of the stack.

To verify the actual drift correction effect of the proposed integrated online correction method (a combination of the FIR algorithm with LIM correction), we performed another stack scan of 100 images with an energy step size of 0.3 eV in the energy range 440 eV–470 eV for the same particle sample as in the Section 3.1. Nine resulting images with 3.6 eV energy intervals were taken from the stack and shown in Fig. 9 ▸. The particle contour (blue outline) of the first image was superimposed on each resulting image to visually display the drift correction effects of the proposed fine correction method.

After the offset of the particle position is calculated for each image using FIR, the residual sample drifts of the above nine energy points after fine correction with the integrated online drift correction method are listed in Table 4 ▸. It can be seen that, with the FIR method included in the drift correction, the sample drifts in the *X* direction are reduced to less than 60 nm and in the *Y* direction reduced to less than 120 nm. In addition, the drift does not monotonically increase with the change of energy, but just randomly fluctuates. From these data, we can calculate the root-mean-square (RMS) error of the *X*-direction drift to be 2.84 nm and the RMS error of the *Y*-direction drift to be 3.85 nm. Therefore, it is highly feasible to accurately correct stack drift online by using the proposed integrated online correction method with FIR included.

At the same time, we analyzed the time requirement of the integrated online drift correction method. According to the experimental measurement results, the time required for Fourier registration between two images is about 100 ms, which is almost negligible compared with the minute-level time required for a STXM scan. Therefore, the integrated online correction method can achieve the sample drift correction with high precision but without affecting the total stack scan time.

## 4. Conclusions

This paper presents an integrated online drift correction method for stack scan in STXM. Without correction, the sample drift will be at the µm level and continuously increase with energy changing. With the proposed online correction method, the sample drift in a stack scan is reduced to less than 120 nm, and its RMS error is less than 4 nm, meaning a high stability of stack scan. Using this method, the time spent on position alignment between two STXM images does not exceed 100 ms, which is negligible compared with the total stacking time. This online correction method significantly reduces the redundant areas required for conventional stack scans, thus greatly decreases the total time of a stack scan and improves the experimental efficiency.

As the method based on FIR is an *a posteriori* correction method, it cannot predict the sample drifts in the subsequent stack process, but only correct the drifts based on the previous scan process. Therefore, although the RMS error of corrected sample offsets using this method is small, the residual offset may still be more than 100 nm. One possible solution to this problem is to scan an additional energy point at the beginning of a stack scan and discard this image during data post-processing. This may allow us to achieve higher precision in sample drift online correction.

## Figures and Tables

**Table 1:** *X*- and *Y*-direction offsets of the sample center in the images of Fig. 2 ▸

Energy (eV)	*X*-direction offset (µm)	*Y*-direction offset (µm)
830	0	0
832.5	0.104	0.311
835	0.047	0.842
837.5	0.084	1.245
840	0.143	1.731
842.5	0.179	2.049

**Table 2:** Sample drifts in *X* and *Y* directions for the two stack scans

	*X* direction (µm)	*Y* direction (µm)
Sample drift with 0.3 eV steps	1.138	1.079
Sample drift with a 30 eV step	1.024	0.413
Difference (drift due to motor thermal drift)	0.114	0.666

**Table 3:** Sample shifts of the stack images relative to the first image without or with rough correction

Energy (eV)	*X*-direction drift (µm)		*Y*-direction drift (µm)	
	No correction	Rough correction	No correction	Rough correction
440.0	0.000	0.000	0.000	0.000
443.6	0.107	0.000	0.184	0.004
447.2	0.234	0.005	0.288	0.104
450.8	0.381	0.035	0.388	0.304
454.4	0.509	0.049	0.592	0.303
458.0	0.660	0.127	0.681	0.398
461.6	0.785	0.166	0.873	0.493
465.2	0.951	0.220	0.971	0.399
468.8	1.059	0.220	1.095	0.304

**Table 4:** Residual sample drifts of the stack images after fine correction with the proposed method

Energy (eV)	*X*-direction drift (µm)	*Y*-direction drift (µm)
440.0	0.000	0.000
443.6	0.060	0.095
447.2	0.031	0.099
450.8	0.035	0.119
454.4	0.026	0.095
458.0	0.036	0.105
461.6	0.026	0.109
465.2	0.046	0.095
468.8	0.036	0.115
